# Extraction Optimization and Effects of Extraction Methods on the Chemical Structures and Antioxidant Activities of Polysaccharides from Snow Chrysanthemum (*Coreopsis Tinctoria*)

**DOI:** 10.3390/polym11020215

**Published:** 2019-01-26

**Authors:** Huan Guo, Qin Yuan, Yuan Fu, Wen Liu, Ya-Hong Su, Hui Liu, Chao-Yi Wu, Li Zhao, Qing Zhang, De-Rong Lin, Hong Chen, Wen Qin, Ding-Tao Wu

**Affiliations:** College of Food Science, Sichuan Agricultural University, Ya’an 625014, Sichuan, China; ghscny@163.com (H.G.); qysicau@163.com (Q.Y.); yuanffuy@163.com (Y.F.); wlsicau@163.com (W.L.); syhsicau@163.com (Y.-H.S.); lhsicau@163.com (H.L.); wcysicau@163.com (C.-Y.W.); zhaoli0608@126.com (L.Z.); zhangqing@sicau.edu.cn (Q.Z.); lindr2018@sicau.edu.cn (D.-R.L.); chenhong945@sicau.edu.cn (H.C.); qinwen@sicau.edu.cn (W.Q.)

**Keywords:** *Coreopsis tinctoria*, polysaccharide, extraction optimization, chemical structure, antioxidant activity

## Abstract

In order to explore snow chrysanthemum polysaccharides (SCPs) as functional food ingredients and natural antioxidants for industrial applications, both microwave-assisted extraction (MAE) and ultrasonic-assisted extraction (UAE) were firstly optimized for the extraction of SCPs. Furthermore, the effects of conventional hot water extraction, UAE, and MAE on the chemical structures and antioxidant activities of SCPs were investigated. The maximum extraction yields of SCPs extracted by UAE (4.13 ± 0.24%) and MAE (4.26 ± 0.21%) were achieved at the optimized extraction parameters as follows: ultrasound amplitude (68%) and microwave power (500 W), ultrasound extraction time (21 min) and microwave extraction time (6.5 min), and ratio of liquid to raw material (42.0 mL/g for UAE and 59.0 mL/g for MAE). In addition, different extraction methods significantly affected the contents of uronic acids, the molecular weights, the molar ratio of constituent monosaccharides, and the degree of esterification of SCPs. SCPs exhibited remarkable DPPH (IC_50_ ≤ 1.702 mg/mL), ABTS (IC_50_ ≤ 1.121 mg/mL), and nitric oxide (IC_50_ ≤ 0.277 mg/mL) radical scavenging activities, as well as reducing power (≥ 80.17 ± 4.8 μg Trolox/mg), which suggested that SCPs might be one of the major contributors toward the antioxidant activities of snow chrysanthemum tea. The high antioxidant activities (DPPH, IC_50_ = 0.693 mg/mL; ABTS, IC_50_ = 0.299 mg/mL; nitric oxide, IC_50_ = 0.105 mg/mL; and reducing power, 127.79 ± 2.57 μg Trolox/mg) observed in SCP-M extracted by the MAE method might be partially attributed to its low molecular weight and high content of unmethylated galacturonic acids. Results suggested that the MAE method could be an efficient technique for the extraction of SCPs with high antioxidant activity, and SCPs could be further explored as natural antioxidants for industrial application.

## 1. Introduction

Oxidative stress is usually caused by reactive oxygen species (ROS) [1]. Elevated level of ROS results in the generation of free radicals which can cause deleterious effects on protein, lipids, and deoxyribonucleic acid (DNA) [2]. Free radicals-induced oxidative stress plays an important role in the pathophysiology of various disease conditions, such as neurodegenerative disorders, cancer, cardiovascular, inflammatory diseases, and aging [3,4]. Antioxidants can alleviate the oxidative stress, which is beneficial for human health. Nowadays, many synthetic antioxidants have been widely used [5]. However, due to the side effects of some synthetic antioxidants, there is an increasing interest in seeking for natural antioxidants. A variety of natural polysaccharides have attracted great interest due to their antioxidant functions, such as scavenging free radicals and reducing oxidative damages [6,7,8]. Polysaccharides can protect body tissue against ROS-induced decline through the radical scavenging activity and immunoregulatory activity [9]. At present, lots of different polysaccharides with excellent antioxidant activities have been isolated from natural sources [8,10].

The flower of *Coreopsis tinctoria* Nutt., also known as “snow chrysanthemum”, is a popular tea material and an important food and medicine dual-purpose plant in China [11]. *C. tinctoria* is native in North America but is now distributed worldwide and is widely cultivated at an altitude of above 3000 m around Kunlun Mountain in the Xinjiang Uygur Autonomous Region of China [12]. Snow chrysanthemum has been traditionally used for the treatment of diabetes, cardiovascular disease, diarrhea, and internal pains and bleeding, etc. [12,13,14,15]. Pharmacological studies have shown that the extracts of snow chrysanthemum possess multiple activities [15], such as hepatoprotective [16,17], cytoprotective [18], antiproliferative [11], and anti-diabetic effects [19], and antioxidant activity is one of the most concerned with [12,20,21,22,23]. Phenolic compounds in ethanol extracts have been identified as natural antioxidants of snow chrysanthemum [20,21,24]. However, chemical structures and antioxidant activities of polysaccharides, which abundant exist in snow chrysanthemum tea [15], have seldom been investigated. Therefore, the investigation of physicochemical characteristics and antioxidant activities of snow chrysanthemum polysaccharides (SCPs) is necessary, which is beneficial to well understand the chemical characters and antioxidant activity of snow chrysanthemum tea (water decoction).

Generally, extraction techniques significantly influence extraction yields, chemical structures, and bioactivities of natural polysaccharides [25,26], which have great effects on the utilization of natural polysaccharides for industrial applications. The traditional hot water extraction (HWE) is the most common method for the extraction of polysaccharides. However, HWE has the disadvantages of high extraction temperature, long extraction time, and low extraction efficiency [27]. So, some physical methods which could facilitate the extraction process have been taken into consideration. The microwave-assisted extraction (MAE) and ultrasonic-assisted extraction (UAE) have been indicated to have higher extraction efficiency than that of HWE [25,26,28]. Indeed, several studies have demonstrated that natural polysaccharides extracted by MAE and UAE exhibit much higher antioxidant activity than that of HWE [25,28,29,30]. However, to the best of our knowledge, the optimization of microwave-assisted extraction and ultrasonic-assisted extraction of SCPs and the effects of different extraction methods on their chemical characteristics and antioxidant activities have seldom been investigated. Therefore, in order to evaluate the effects of different extraction methods on the physicochemical characteristics and antioxidant activities of SCPs and further explore SCPs as functional food ingredients and natural antioxidants for industrial applications, both MAE and UAE were firstly optimized for the extraction of SCPs, and then the chemical structures and antioxidant activities of SCPs extracted by different methods (MAE, UAE, and HWE) were systemically investigated and compared.

## 2. Material and Methods

### 2.1. Material and Chemicals

The flower of snow chrysanthemum (*C. tinctoria*) was purchased from a local market in Ya’an, China. Samples were dried at the temperature of 45 °C for 2 days. Subsequently, the samples were ground to pass through a 60 mesh sieve and stored at −20 °C for further analysis.

Trifluoroacetic acid, rhamnose (Rha), mannose (Man), glucuronic acid (GlcA), galacturonic acid (GalA), glucose (Glc), galactose (Gal), xylose (Xyl), arabinose (Ara), 1-phenyl-3-methyl-5-pyrazolone (PMP), *m*-hydroxydiphenyl, griess reagent, sodium nitroprusside (SNP), 2,2-diphenyl-1-(2,4,6-trinitrophenyl) hydrazyl (DPPH), 2,2′-azino-bis(3-ethylbenzothiazoline-6-sulphonic acid) (ABTS), vitamin C, butylated hydroxytoluene (BHT), and 6-hydroxy-2,5,7,8-tetramethyl chroman-2-carboxylic acid (Trolox) were purchased from Sigma-Aldrich (St. Louis, MO, USA). All other reagents and chemicals used were of analytical grade.

### 2.2. Extraction of Polysaccharides from Snow Chrysanthemum

#### 2.2.1. Hot Water Extraction

The hot water extraction (HWE) was performed according to a previously reported method with some modifications [31]. Briefly, the snow chrysanthemum powders (1.0 g) were firstly refluxed with 10 mL of 80% (*v*/*v*) ethanol at 80 °C for 2 h to remove most of the small molecules. Subsequently, the snow chrysanthemum polysaccharides (SCPs) were extracted twice with 30 mL of deionized water at 90 °C for 2 h. After centrifugation at 4000× *g* for 15 min (Heraeus Multifuge X3R Centrifuge, Thermo Fisher scientific, Waltham, MA, USA), the supernatants were combined and concentrated to 1/3 of the origin volume by a rotary evaporator under a vacuum at 60 °C. Furthermore, the supernatants were precipitated with three volumes of 95% ethanol (*v*/*v*) overnight at 4 °C. The precipitations were washed with 70% ethanol (*v*/*v*) for three times and then dissolved in deionized water. Finally, the crude snow chrysanthemum polysaccharides (SCP-W) were freeze dried and stored at −20 °C for further analysis.

#### 2.2.2. Ultrasonic-Assisted Extraction

Both the single-factor experimental design and Box–Behnken experimental design were applied for the optimization of the ultrasonic-assisted extraction (UAE) conditions. The SCPs were extracted by ultrasonic-assisted extraction with an Ultrasonic Processor (650 W, 24 kHz, Scientz, Ningbo, China) at room temperature. Briefly, the snow chrysanthemum powder (1.0 g) was firstly refluxed with 10 mL of 80% (*v*/*v*) ethanol at 80 °C for 2 h to remove most of the small molecules. Subsequently, the extract residue was extracted with deionized water by UAE, and the effects of the ultrasound amplitudes (20%, 40%, 60%, 80%, and 100%), extraction time (5, 10, 15, 20, and 25 min), and ratio of water to raw material (20, 30, 40, 50, and 60 mL/g) on the yield of SCPs were investigated using a single-factor experimental design. Finally, the crude snow chrysanthemum polysaccharides (SCP-U) were obtained according to the same treatment processes as described in Section 2.2.1.

Furthermore, based on the results of the single-factor experiments, a three-level Box–Behnken experimental design (BBD) with three factors was applied to optimize the UAE conditions. The ultrasound amplitude (*X*_11_, amplitude %), extraction time (*X*_12_, min), and ratio of water to raw material (*X*_13_, mL/g) were preferred for the independent variables. According to the BBD design, 17 experimental runs with 1 block and 5 centre points were performed. The variables and their levels, with both coded and actual values were presented in Table 1. The obtained data was analyzed by the statistical function of the Design Expert software 8.0.5 (Stat-Ease Inc., Minneapolis, MN, USA). The significance of the model was evaluated by the analysis of variance (ANOVA). Experimental data from BBD were explained by the second-order polynomial model as follows [32]:

(1) $$Y=A_{0}+\overset{3}{\underset{i=1}{\sum }}A_{i}X_{i}+\overset{3}{\underset{i=1}{\sum }}A_{ii}X^{2}{}_{i}+\overset{2}{\underset{i=1}{\sum }}\overset{3}{\underset{j=i+1}{\sum }}A_{ij}X_{i}X_{j}$$

where Y is the predicted response; *X_i_* and *X_j_* are different variables (*i* ≠ *j*); *A*_0_, *A_i_*, *A_ii_*, and *A_ij_* are regression coefficients for the intercept, linearity, square, and interaction, respectively.

#### 2.2.3. Microwave-Assisted Extraction

Both the single-factor experimental design and Box–Behnken experimental design were also applied for the optimization of the microwave-assisted extraction (MAE) conditions. Briefly, the snow chrysanthemum powder (1.0 g) was firstly refluxed with 10 mL of 80% (*v*/*v*) ethanol at 80 °C for 2 h to remove most of the small molecules. Subsequently, the extract residue was extracted with deionized water by MAE (MKJ-J1-3, Qingdao Makewave Microwave Applied Technology Co., Ltd., Shandong, China), and the effects of the microwave power (240, 320, 400, 480, and 560 W), extraction time (2, 4, 6, 8, and 10 min), and ratio of water to raw material (30, 40, 50, 60, and 70 mL/g) on the yield of SCPs were investigated using a single-factor experimental design. Finally, the crude snow chrysanthemum polysaccharides (SCP-M) were obtained according to the same treatment processes as described in Section 2.2.1.

Furthermore, based on the results of the single-factor experiments, a three-level Box–Behnken experimental design (BBD) with three factors was also applied to optimize the MAE conditions. The microwave power (*X*_21_, W), extraction time (*X*_22_, min), and ratio of water to raw material (*X*_23_, mL/g) were preferred for the independent variables. The variables and their levels, with both coded and actual values, were also presented in Table 1. Statistical analysis was performed the same as described in Section 2.2.2.

### 2.3. Characterization of Polysaccharides from Snow Chrysanthemum

#### 2.3.1. Chemical Composition Analysis

The chemical compositions, such as the content of total polysaccharides, content of uronic acids, and content of proteins, were analyzed by colorimetric methods. The content of total polysaccharides in SCPs was determined by the phenol-sulfuric acid method with a mixture standard [33]. In order to reduce the interference of different monosaccharides on the response of the phenol-sulfuric acid method, a mixture standard was prepared by 40% GalA, 30% Ara, and 30% Gal according to the constituent monosaccharides in SCPs determined by a high-performance liquid chromatography (HPLC, ThermoFisher scientific, Waltham, MA, USA). The content of uronic acids in SCPs was determined by using the *m*-hydroxydiphenyl method with GalA as a standard [34]. The content of proteins in SCPs was determined by using Bradford’s method with bovine serum albumin as a standard [35].

#### 2.3.2. Determination of Molecular Weights

The absolute molecular weights (*M_w_*) and polydispersities (*M_w_*/*M_n_*) of SCPs were measured by high-performance size-exclusion chromatography coupled with multi-angle laser light scattering and refractive index detector (HPSEC-MALLS-RID, Wyatt Technology Co., Santa Barbara, CA, USA) according to our previously reported method with minor modifications [36]. In brief, HPSEC-MALLS-RID measurements were carried out on a multi-angle laser light scattering detector (DAWN HELEOS, Wyatt Technology Co., Santa Barbara, CA, USA) with an Agilent 1260 series LC system (Agilent Technologies, Palo Alto, CA, USA). TSK-Gel G5000PWXL (300 mm × 7.8 mm, i.d.) and TSK-Gel G3000PWXL (300 mm × 7.8 mm, i.d.) were used in series at 30 °C. The MALLS instrument was equipped with a He–Ne laser (λ = 658 nm). An Optilab rEX refractometer (DAWN EOS, Wyatt Technology Co., Santa Barbara, CA, USA) was simultaneously connected. The mobile phase was 0.9% NaCl aqueous solution at a flow rate of 0.5 mL/min. The sample concentration was about 1.0 mg/mL. An injection volume of 100 μL was used. The *dn/dc* value of SCPs was selected as 0.15 mL/g according to a previous study [36]. The Astra software (version 6.1.2, Wyatt Technology Co., Santa Barbara, CA, USA) was utilized for data acquisition and analysis.

#### 2.3.3. Determination of Constituent Monosaccharides

Constituent monosaccharides of SCP-W, SCP-U, and SCP-M were measured by HPLC analysis according to a previously reported method with some modifications [37]. Briefly, each sample (4.0 mg) was hydrolyzed with 2.0 M trifluoracetic acid (2.0 mL) at 95 °C for 10 h. After hydrolysis, the hydrolysates were evaporated to dryness by a rotary evaporator under a vacuum and washed with methanol to remove the residue of the trifluoroacetic acid. Subsequently, the dried hydrolyzates were dissolved in 1 mL of water for subsequent derivatization.

Furthermore, 50 µL of hydrolyzates was mixed with 50 µL of 0.6 M sodium hydroxide and 100 µL of 0.5 M PMP methanol solution. The mixture was incubated at 70 °C for 100 min with continuous shaking. Then, 80 µL of 0.3 M hydrochloric acid solution was used to neutralize the mixture, and the mixture was diluted to 1 mL with pure water. One mL of chloroform was added. After vigorous shaking and layering, the organic phase was discarded. The operation was performed in triplicate, and finally, the solution was passed through a 0.22 µm organic syringe filter for HPLC analysis. A standard solution, containing Rha, Man, GlcA, GalA, Glc, Gal, Xyl, and Ara, was derivatized as described above. Finally, The PMP derivatives were analyzed by a Dionex UltiMate 3000 HPLC system (ThermoFisher scientific, Waltham, MA, USA) with a ZORBAX Eclipse XDB-C18 column (4.6 × 250 mm i.d. 5 µm, Agilent Technologies Inc., CA, USA) and a diode array detector (DAD, ThermoFisher scientific, Waltham, MA, USA). The PMP derivative (20 µL) was injected into the HPLC system at the operation temperature of 30 °C and eluted with a mixture of 0.1 M phosphate buffer solution (pH = 6.7) and acetonitrile (83:17, *v*/*v*) at a flow rate of 1.0 mL/min. The wavelength of the DAD was set at 245 nm.

#### 2.3.4. Fourier Transform Infrared (FT-IR) Spectroscopy Analysis

Each sample (1.0 mg) was mixed with 100 mg of dried KBr and pressed into disk for the analysis. The IR spectra were recorded in the frequency range of 4000–500 cm^−1^ with a Nicolet iS 10 FT-IR (ThermoFisher scientific, Waltham, MA, USA). Furthermore, the esterification degrees (DE) of SCP-W, SCP-U, and SCP-M were determined from the FT-IR spectra according to previously reported methods [38,39]. The determination of DE was based on the band areas at 1700–1750 cm^−1^ (esterified uronic acids) and 1600–1630 cm^−1^ (free uronic acids). DE was calculated according to the equation as follows:

(2) $$\mathrm{DE}\left(\%\right)=\left(\frac{A_{1741}}{A_{1741}+A_{1621}}\right)\times 100$$

### 2.4. Evaluation of Antioxidant Activities of Polysaccharides from Snow Chrysanthemum

#### 2.4.1. DPPH Radical Scavenging Activity

The DPPH radical scavenging activities of SCP-W, SCP-U, and SCP-M were determined according to our previously reported method with minor modifications [40]. Briefly, 200 µL of 0.35 mM DPPH solution was mixed with 20 µL of each sample at different concentrations (0.2, 0.4, 0.6, 0.8, 1.0, and 1.2 mg/mL) or deionized water as a negative control in a 96-well microplate. Then, the mixture was incubated at 37 °C for 30 min in the dark, and the absorbance was measured at 517 nm. BHT was used as a positive control. The DPPH radical scavenging activity (%) was calculated as follows:

(3) $$\mathrm{DPPH}\;\mathrm{radical}\;\mathrm{scavenging}\;\mathrm{activity}\;\left(\%\right)=\left(1-\frac{A_{\mathrm{sample}}-A_{\mathrm{control}}}{A_{\mathrm{blank}}}\right)\times 100\%$$

where A_sample_ is the absorbance of the mixture of sample and DPPH work solution; A_control_ is the absorbance of the mixture of deionized water and sample; and A_blank_ is the absorbance of the mixture of deionized water and DPPH work solution.

#### 2.4.2. ABTS Radical Cation Scavenging Activity

The ABTS radical cation scavenging activities of SCP-W, SCP-U, and SCP-M were also measured according to our previously reported method with minor modifications [40]. Briefly, the ABTS radical cation solution was generated by the interaction of 7 mM ABTS solution and 2.45 mM aqueous potassium persulfate at room temperature for at least 16 h in the dark. The ABTS radical cation solution was diluted with phosphate buffer (0.2 M, pH 7.4) to an absorbance of 0.750 ± 0.02 at 734 nm. Then, 200 μL of ABTS radical cation working solution was mixed with 20 μL of each sample at different concentrations (0.1, 0.2, 0.3, 0.4, 0.5, and 0.6 mg/mL) or phosphate buffer as a negative control in a 96-well microplate to react at 30 °C for 20 min. The absorbance at 734 nm was measured. BHT was used as a positive control, and the ABTS radical scavenging activity was calculated as follows:

(4) $$\mathrm{ABTS}\;\mathrm{radical}\;\mathrm{scavenging}\;\mathrm{activity}\;\left(\%\right)=\left(1-\frac{A_{\mathrm{sample}}-A_{\mathrm{control}}}{A_{\mathrm{blank}}}\right)\times 100\%$$

where A_sample_ is the absorbance of the mixture of sample and ABTS work solution; A_control_ is the absorbance of the mixture of deionized water and sample; and A_blank_ is the absorbance of the mixture of deionized water and ABTS work solution.

#### 2.4.3. Nitric Oxide Radical Scavenging Activity

The nitric oxide (NO) radical scavenging activities of SCP-W, SCP-U, and SCP-M were measured according to a previously reported methods with some modifications [41]. Briefly, 50 μL of 10 mM sodium nitroprusside (SNP, prepared in 200 mM phosphate buffer (PBS), pH = 6.6) was mixed with 450 μL of each sample at different concentrations (0.1, 0.2, 0.3, 0.4, 0.5, and 0.6 mg/mL) and incubated at 25 °C for 3 h in front of a visible polychromatic light source. Then, 250 μL of Griess reagent (1% sulfanilamide and 0.1% naphthylethylenediamine dihydrochloride in 2% phosphoric acid) was added into the mixture. Finally, the absorbance was measured at 540 nm, and vitamin C was used as a positive control. The nitric oxide radical scavenging activity was calculated as follows:

(5) $$\mathrm{NO}\;\mathrm{radical}\;\mathrm{scavenging}\;\mathrm{activity}\;\left(\%\right)=\left(1-\frac{A_{\mathrm{sample}}-A_{\mathrm{control}}}{A_{\mathrm{blank}}}\right)\times 100\%$$

where A_sample_ is the absorbance of the mixture of the sample, the SNP solution, and the Griess reagent; A_control_ is the absorbance of the mixture of deionized water and the sample; and A_blank_ is the absorbance of the mixture of deionized water, the SNP solution, and the Griess reagent.

#### 2.4.4. Reducing Power

The reducing power was determined according to our previously reported method with minor modifications [40]. Briefly, an aliquot of 100 µL of each sample at different concentrations (0.2, 0.4, 0.6, 0.8, 1.0, and 1.2 mg/mL) was mixed with 100 µL of potassium ferricyanide (1%, *w*/*v*) in PBS (pH 6.8, 20 mM). After the mixture was incubated at 50 °C for 20 min, 100 µL of trichloroacetic acid (10%, *w*/*v*) was added, followed by centrifugation at 3000× *g* for 10 min. The supernatant (100 µL) was mixed with 100 µL of distilled water and 20 µL of ferric chloride (0.1%, *w*/*v*). The absorbance was measured at 700 nm after a 30 min incubation. The blank control contained all the reagents except the sample. BHT was used as the standard, and the reducing powers of the SCPs were expressed as micrograms of Trolox equivalent per milligram of SCPs (μg Trolox/mg).

### 2.5. Statistical Analysis

All experiments were conducted in triplicate, and data were expressed in means ± standard deviations. Statistical analysis was performed using Origin 9.0 software (OriginLab Corporation, Northampton, Mass., USA). Statistical significances were carried out by one-way analysis of variance (ANOVA), followed by Duncan’s test. Values of *p* < 0.05 were considered as statistically significant.

## 3. Results and Discussions

### 3.1. Extraction Optimization of Polysaccharides from Snow Chrysanthemum

#### 3.1.1. Ultrasonic-Assisted Extraction of SCPs

**Figure 1:**
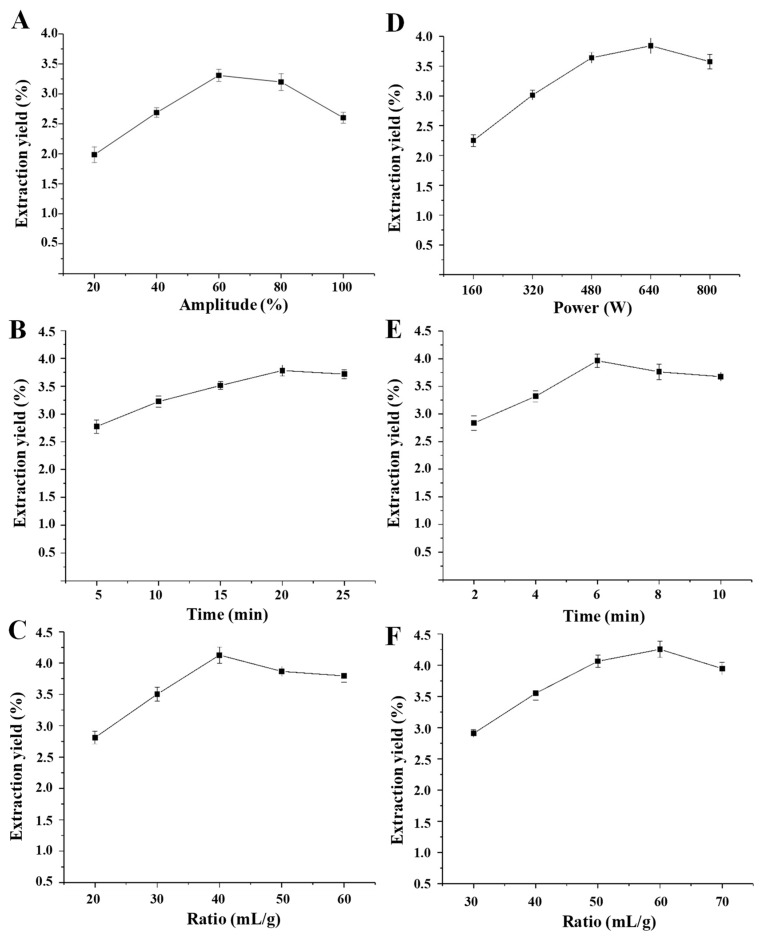
The effects of different extraction parameters of ultrasonic-assisted extraction and microwave-assisted extraction on the yields of polysaccharides extracted from snow chrysanthemum: (**A**) the ultrasound amplitude, (**B**) the ultrasound extraction time, and (**C**) the ratio of water to raw material of ultrasonic-assisted extraction; (**D**) the microwave power, (**E**) the microwave extraction time, and (**F**) the ratio of water to raw material of microwave-assisted extraction.

The factors of ultrasound amplitude (power), ultrasound extraction time, and ratio of water to raw material significantly affect the yield of polysaccharides extracted by UAE [32]. Therefore, the single-factor experimental design was firstly applied for the optimization of UAE conditions. The effects of ultrasound amplitude, ultrasound extraction time, and ratio of water to raw material on the yields of SCPs are shown in Figure 1. Briefly, the extraction yields of SCPs increased with the increase of the ultrasound amplitude from 20% to 60% and reached a maximum yield at 60% (Figure 1A). However, after 60%, there was a significant decrease in the yield of SCPs, which might be due to the degradation of SCPs under excessive ultrasonic power [42]. The extraction yields of SCPs also increased with the increase of extraction time from 5 min to 20 min and reached a maximum yield at 20 min (Figure 1B). When the extraction time continued to increase, the extraction yield no longer changed. In addition, the extraction yields of SCPs increased quickly with the increase of the ratio of water to raw material from 20 mL/g to 40 mL/g (Figure 1C). However, the extraction yield decreased slowly with the continued increase of the ratio of water to raw material, which is in accordance with previous studies [32]. Finally, results showed that the optimal ultrasound amplitude, the optimal extraction time, and the optimal ratio of water to raw material were determined to be 60%, 20 min, and 40 mL/g according to the single-factor experimental design, respectively.

Furthermore, Table 1 showed the BBD matrix and the experimental data. Using multiple regression analysis, Design Expert 8.0.5 generated a second-order polynomial equation to express the relationship between the process and response. The final equation in terms of the coded factors was as follows:

(6) $$\begin{array}{c} Y_{1}=4.08+0.32X_{11}+0.041X_{12}+0.15X_{13}+0.11X_{11}X_{12}-0.11X_{11}X_{13}-0.043X_{12}X_{13}\\ -0.51X_{11}^{2}-0.32X_{12}^{2}-0.20X_{13}^{2} \end{array}$$

where Y_1_ represents the extraction yield and *X*_11_, *X*_12_, and *X*_13_ are the ultrasound amplitude, extraction time, and ratio of water to raw material, respectively.

The statistical significance of the second-order polynomial equation was analyzed by using one-way ANOVA. As shown in Table 2, the quadratic regression model has a high *F*-value (121.60) and a very low *p*-value (*p* < 0.0001), which indicated that the fitness of the model is highly significant [43]. The lack of fit *F*-value of 4.79 and *p*-value of 0.0821 (*p* > 0.05) implied that the lack of fit was not significant, which indicated that the model equation is adequate for predicting the yield of SCPs under the conditions of any combination of the variables’ values [44]. In addition, the low value of the coefficient variation (*C.V.*, 1.45%) and the high value of the adeq. precision (32.118) indicated that this model had good precision and reliability [45]. The coefficient of determination (*R*^2^) and adjusted coefficient of determination (*R*^2^*_adj_*) were 0.9936 and 0.9855, respectively, which indicated that this polynomial model had adequate accuracy and general applicability [46]. Moreover, the linear coefficients (*X*_11_ and *X*_13_), interaction coefficients (*X*_11_*X*_12_ and *X*_11_*X*_13_), and quadratic term coefficients (*X*_11_, *X*_12_, and *X*_13_) were significant (*p* < 0.05), while the linear coefficients (*X*_12_) and interaction coefficients (*X*_12_*X*_13_) had no significant influence (*p* > 0.05) on the extraction yield.

**Figure 2:**
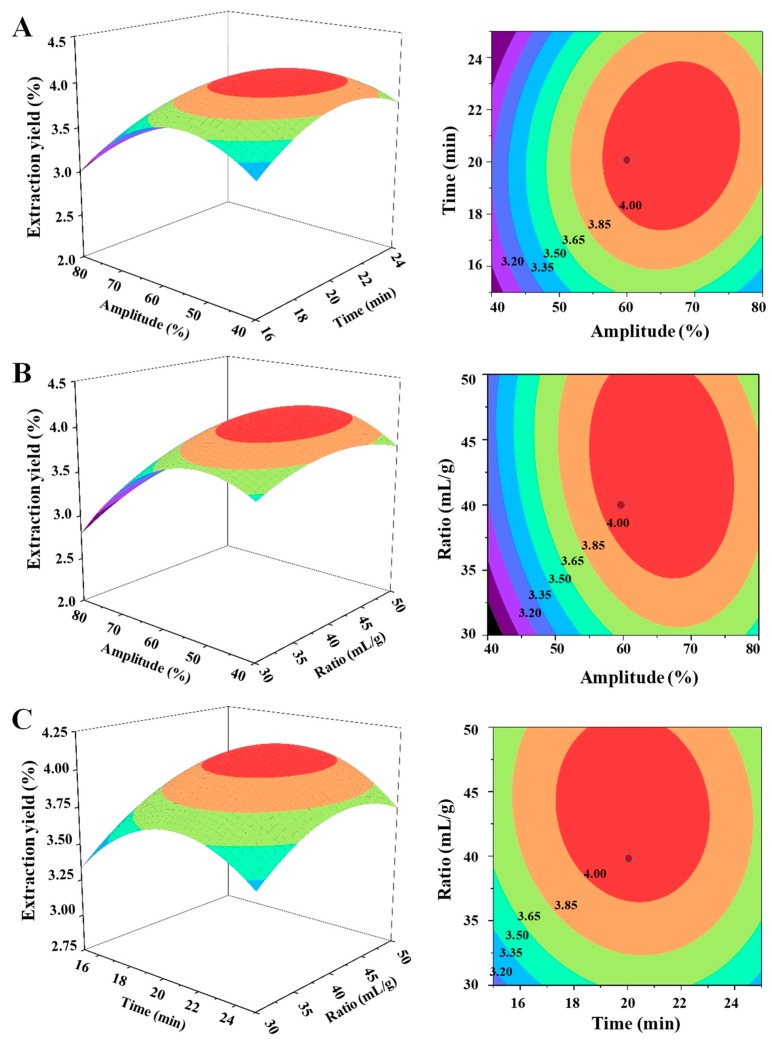
The three-dimensional response surface (left) and two-dimensional contour (right) plots of ultrasonic-assisted extraction: (**A**) ultrasound amplitude and ultrasound extraction time; (**B**) ultrasound amplitude and the ratio of water to raw material; and (**C**) ultrasound extraction time and the ratio of water to raw material.

The predicted models were presented in three-dimensional (3D) response surface plots and two-dimensional contour plots as shown in Figure 2. Generally, the response surface with circular contour plot indicates that the interaction between the corresponding variables is negligible, whereas an elliptical contour plot indicates that the interaction between the corresponding variables is significant [47,48]. In this study, it was clear that the interactions between ultrasound amplitude and extraction time and between ultrasound amplitude and the ratio of water to raw material were significant. However, the interaction between extraction time and the ratio of water to raw material was not significant. Furthermore, the model predicted the maximum extraction yield (4.13%) could be obtained under the following optimal extraction conditions: ultrasound amplitude of 67.81%, ultrasound extraction time of 20.97 min, and ratio of water to raw material of 42.02 mL/g. Considering the operability in the actual processing procedure, the verification experiment was carried out under the following conditions: ultrasound amplitude of 68.0%, ultrasound extraction time of 21.0 min, and ratio of water to raw material of 42.0 mL/g. Under these optimal UAE conditions, the actual extraction yield of SCPs was 4.13 ± 0.24% (n = 3), which was in good agreement with the predicted value. The extraction yield of SCPs extracted by UAE (4.13%) in the present study was much higher than that of SCPs extracted by hot water extraction (1.4%) in a previous study [49]. In addition, the extraction time of 21.0 min for UAE was much shorter than that of hot water extraction (6 h), which suggested that the optimized UAE method was more efficient than the hot water extraction. Furthermore, although the extraction yield of SCPs extracted by UAE (4.13%) was lower than that of SCPs extracted by the ultrasound-assisted combined enzymolysis (USCE) method (9.8%), the total polysaccharide content of SCPs extracted by UAE (79.27%) was much higher than that of SCPs extracted by USCE (28.6%) [11]. Compared with the USCE method, the optimized UAE method in the present study was free of all kinds of enzymes and the extraction time was much shorter [11].

#### 3.1.2. Microwave-Assisted Extraction of SCPs

The microwave power, extraction time, and ratio of water to raw material are significant parameters to affect the extraction yield of polysaccharides [50]. Thus, the single-factor experimental design was firstly applied for the optimization of MAE conditions. The effects of microwave power, extraction time, and the ratio of water to raw material on the yield of SCPs were shown in Figure 1. Briefly, the extraction yield of SCPs increased with the increase of the microwave power from 160 W to 640 W and reached a maximum yield at 640 W (Figure 1D). However, there was a slight decrease in the yield of SCPs after 640 W, which might be attributed to the degradation of polysaccharides under too high microwave power [45]. The extraction yield of SCPs also increased with the increase of extraction time from 2 min to 6 min and reached a maximum yield at 6 min (Figure 1E). When the extraction time increased further, the extraction yield slightly decreased. This could be implied that excessive extraction time with microwave irradiation lead to the degradation of polysaccharides [45]. Furthermore, the extraction yield of SCPs increased with the increase of the ratio of water to raw material from 30 mL/g to 60 mL/g and reached a maximum yield at 60 mL/g. Finally, results showed that the optimal microwave power, the optimal extraction time, and the optimal ratio of water to raw material were determined to be 640 W, 6 min, and 60 mL/g according to the single-factor experimental design, respectively. Furthermore, Table 1 also summarized the BBD matrix and the experimental data for the MAE method. By applying a multiple regression analysis, a final second-order polynomial equation in terms of coded values was obtained as follows:

$$\begin{array}{c} Y_{2}=4.25+0.18X_{21}+0.22X_{22}+0.037X_{23}-0.015X_{21}X_{22}+0.022X_{21}X_{23}-0.10X_{22}X_{23}-0.31X_{21}^{2}\\ -0.48X_{22}^{2}-0.24X_{23}^{2} \end{array}$$

where Y_2_ represents the extraction yield and *X*_21_, *X*_22_, and *X*_23_ are the microwave power, extraction time, and ratio of water to raw material, respectively.

**Figure 3:**
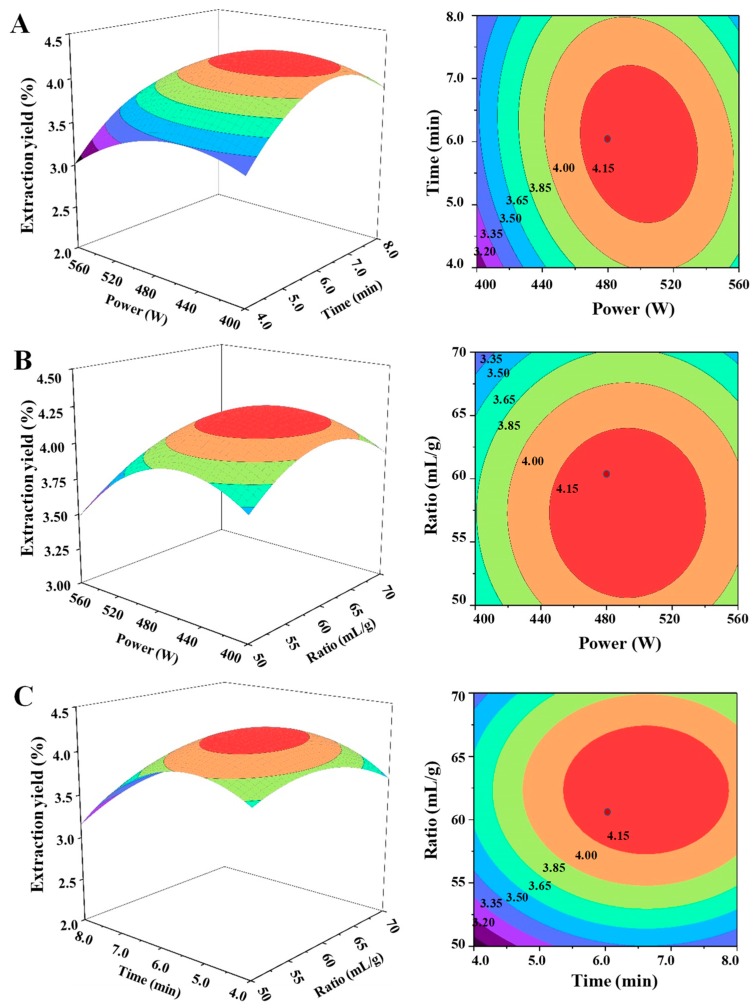
The three-dimensional response surface (left) and two-dimensional contour (right) plots of microwave assisted extraction: (**A**) microwave power and extraction time; (**B**) microwave power and the ratio of water to raw material; and (**C**) extraction time and the ratio of water to raw material.

As shown in Table 2, the quadratic regression model for teh MAE method also had a high *F*-value (541.05) and a very low *p*-value (*p* < 0.0001), which indicated that the fitness of the model is highly significant [43]. Moreover, the lack of fit *F*-value of 5.48 and *p*-value of 0.0670 (*p* > 0.05) also implied that the lack of fit was not significant, which indicated that the model equation is also adequate for predicting the yield of SCPs extracted by MAE. In addition, the low value of the coefficient variation (*C.V.*, 0.60%) and the high value of the adeq. precision (69.468) also indicated that this model had good precision and reliability. The coefficient of determination (*R*^2^) and adjusted coefficient of determination (*R*^2^*_adj_*) were 0.9986 and 0.9867, respectively, which also indicated that this polynomial model had adequate accuracy and general applicability. Moreover, the linear coefficients (*X*_21_, *X*_22_, and *X*_23_), interaction coefficient (*X*_22_*X*_23_), and quadratic term coefficients (*X*_21_, *X*_22_, and *X*_23_) were significant (*p* < 0.05), while the interaction coefficients (*X*_21_*X*_22_ and *X*_21_*X*_23_) had no significant influence (*p* > 0.05) on the extraction yield. In addition, the 3D response surface and 2D contour plots of MAE were shown in Figure 3. The results showed that the interactions between microwave power and extraction time and between microwave power and the ratio of water to raw material were not significant. However, the interaction between extraction time and the ratio of water to raw material was significant. Furthermore, the model predicted the maximum extraction yield (4.29%) could be obtained under the following optimal extraction conditions: microwave power of 497.76 W, extraction time of 6.44 min, and ratio of water to raw material of 58.67 mL/g. To validate the adequacy of the model equation, a verification experiment was carried out under the following conditions: microwave power of 500 W, microwave extraction time of 6.5 min, and ratio of water to raw material of 59.0 mL/g. The actual extraction yield of 4.26 ± 0.21% (n = 3) was obtained, which was in accordance with the predicted value. The extraction yields of SCPs extracted by the optimized MAE and the optimized UAE methods were similar. Compared with the UAE method as described above, the extraction time of MAE was much shorter, but the ratio of water to raw material of MAE was relatively high. Indeed, the extraction efficient of the optimized MAE method was much higher than that of the hot water extraction and the USCE method in previous studies [11,49]. The results suggested that the optimized MAE method was efficient for the extraction of polysaccharides from snow chrysanthemum.

### 3.2. Physicochemical Characteristics of SCPs

#### 3.2.1. Chemical Composition of SCPs

As shown in Table 3, the extraction yields of SCP-W, SCP-U, and SCP-M were similar, which were determined to be 3.98 ± 0.22 %, 4.13 ± 0.24 %, and 4.26 ± 0.21 %, respectively. The results indicated that the HWE, UAE, and MAE had no significant effects on the extraction yields of SCPs under their optimal extraction conditions as abovementioned. However, considering the extraction time and extraction temperature of HWE, UAE, and MAE, both UAE and MAE could be better than HWE [25,30]. The total polysaccharide contents of SCP-W, SCP-U, and SCP-M were similar, which were determined to be 80.15 ± 0.54 %, 79.27 ± 0.42 %, and 82.62 ± 1.09%, respectively. A few proteins were detected in SCP-W, SCP-U, and SCP-M, which ranged from 1.36% to 3.32%. Furthermore, the highest uronic acids content of 40.88% was observed in SCP-W, followed by lower content of 34.45% in SCP-U, and the lowest content of 30.45% in SCP-M. The results suggested that different extraction methods had significant effects on the chemical structures of polysaccharides [25,28]. Indeed, the lower content of uronic acids in SCP-M might be attributed to the degradation of SCPs under microwave treatment.

#### 3.2.2. Molecular Weights and Constituent Monosaccharides of SCPs

**Figure 4:**
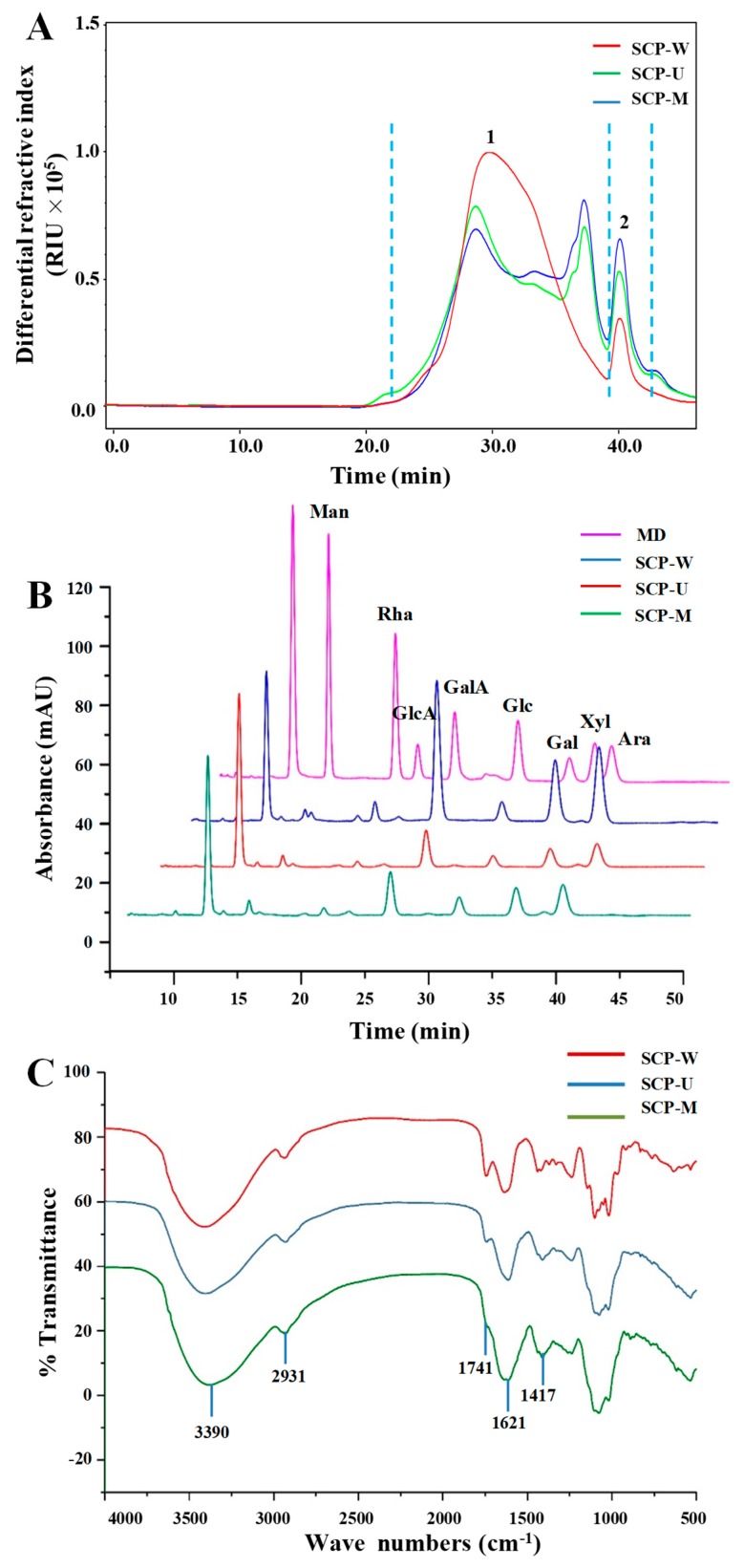
(**A**) High-performance size-exclusion chromatography (HPSEC) chromatograms, (**B**) high-performance liquid chromatography (HPLC) profiles, and (**C**) fourier transform infrared (FT-IR) spectra of SCP-W, SCP-U, and SCP-M; SCP-W, hot water extraction of snow chrysanthemum polysaccharides; SCP-U, ultrasonic assisted extraction of snow chrysanthemum polysaccharides; SCP-M, microwave assisted extraction of snow chrysanthemum polysaccharides; MD, mixed standard of monosaccharides; Man, mannose; Rha, rhamnose; GlcA, glucuronic acid; GalA, galacturonic acid; Glc, glucose; Gal, galactose; Xyl, xylose; and Ara, arabinose.

It is considered that the bioactivities of natural polysaccharides are closely correlated to their molecular weights and constituent monosaccharides [51]. Therefore, the effects of different extraction methods on the molecular weights and constituent monosaccharides of SCPs were investigated. Briefly, Figure 4A showed the HPSEC-RID chromatograms of SCP-W, SCP-U, and SCP-M. Two polysaccharide fractions (Figure 4A, fraction 1 and 2) were detected in SCP-W, SCP-U, and SCP-M. Indeed, a sharp degradation of fraction 1 was detected in the HPSEC chromatograms of SCP-U and SCP-M. Additionally, the molecular weight of fraction 2 could not be precisely determined due to the relatively poor resolution of the column and the co-elution of various different molecules from 38 min to 42 min. Therefore, the molecular weights of polysaccharide fraction 1 in SCP-W, SCP-U, and SCP-M were summarized in Table 3. As shown in Table 3, the molecular weights of polysaccharide fraction 1 in SCPs ranged from 4.336 × 10^5^ Da to 5.719 × 10^5^ Da, which were much higher than that of polysaccharides isolated from *Chrysanthemum morifolium* (a famous chrysanthemum tea) [1,52]. The results showed that the molecular weights of SCP-U extracted by UAE were similar with those of SCP-M extracted by MAE. However, the molecular weight of SCP-W extracted by HWE was significantly (*p* < 0.05) higher than those of SCP-U and SCP-M. The results suggested that both the UAE and MAE methods could degrade the molecular weights of SCPs. Similar studies have shown that the molecular weights of polysaccharides extracted by UAE and MAE are lower than that of conventional HWE [25,26,28]. Furthermore, the polydispersities of polysaccharide fraction 1 in SCP-W, SCP-U, and SCP-M were determined to be 1.52, 1.81, and 1.82, respectively, which further indicated that the molecular weights of SCPs were degraded under ultrasonic-assisted extraction and microwave-assisted extraction.

Furthermore, Figure 4B showed that the HPLC-UV profiles of SCP-W, SCP-U, and SCP-M were similar. The results showed that constituent monosaccharides in SCP-W, SCP-U, and SCP-M were similar, which were measured as Glc, GalA, Gal, Ara, Rha, Man, GlcA, and Xyl. The major constituent monosaccharides (GalA, Gal, and Ara) in SCPs were similar with previous studies [11,49], which suggested that homogalacturonan (HG) existed in snow chrysanthemum polysaccharides. As shown in Table 3, the molar rations of Glc, GalA, Gal, Ara, Rha, Man, GlcA, and Xyl in SCP-W, SCP-U, and SCP-M were different, which were determined to be about 1.00:6.37:5.22:4.76:0.47:0.19:0.12:0.05, 1.00:2.75:2.55:2.41:0.22:0.30:0.11:0.04, and 1.00:2.04:2.41:1.94:0.19:0.22:0.09:0.05, respectively. Results suggested that the UAE and MAE methods had little effect on the types of constituent monosaccharides in SCPs but significantly affected their molar ratios. Similar studies have shown that different extraction techniques affect the monosaccharide compositions of polysaccharides [25,30].

#### 3.2.3. FT-IR Spectra and Degree of Esterification of SCPs

The FT-IR spectra were used for the determination of the structure features of SCPs. As shown in Figure 4C, the FT-IR spectra of SCP-W, SCP-U, and SCP-M were similar. The intense and broad bands around 3200 and 3600 cm^−1^ are the characteristic bands of hydroxyl groups, and the broad band at 3390 cm^−1^ is due to the stretching vibration of hydroxyl group [30]. The broad band at 2931 cm^−1^ is assigned to the C–H asymmetric stretching vibration [30]. The absorption band at 1741 cm^−1^ is the C=O stretching vibration of esterified groups [53]. Furthermore, the intense peak that appeared at 1621 cm^−1^ is the C=O asymmetric stretching of COO^−^, suggesting the existence of uronic acids [53]. The band at 1417 cm^−1^ is attributed to the bending vibration of C–H or O–H [54]. Typical protein band at 1651 cm^−1^ and 1555 cm^−1^ were not detected, which indicated the low amount of protein in SCPs. Furthermore, the effects of different extraction methods on the degree of esterification (DE) of SCPs were also investigated by FT-IR spectroscopy analysis. The significantly highest DE value of 33.07% was observed in SCP-W, followed by the lower DE value of 20.67% in SCP-U, and the lowest DE value of 6.54% in SCP-M. The results suggested that the MAE method significantly affected the DE of SCPs. Previous studies have also indicated that the low DE is observed in pectins extracted under harsh extraction conditions (such as high temperature, high microwave power, and long microwave extraction time) [55,56].

### 3.3. Antioxidant Activities of SCPs

**Figure 5:**
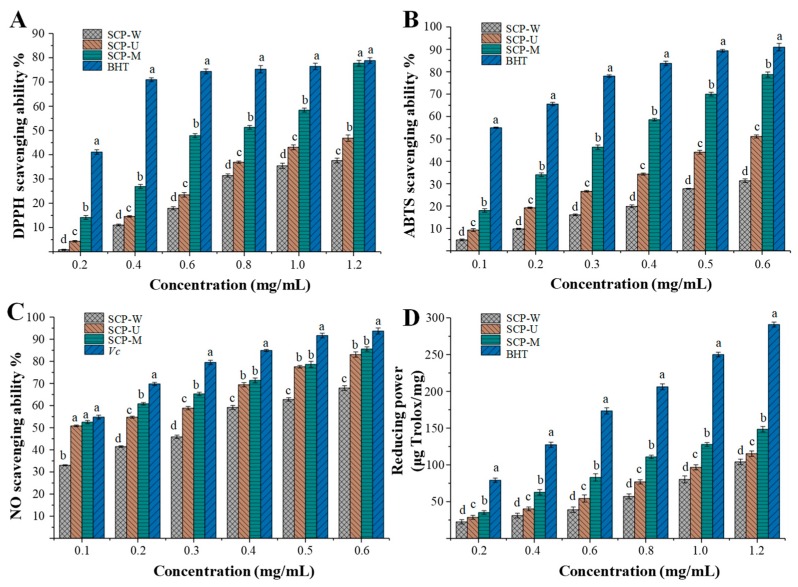
(**A**) DPPH radical scavenging activity, (**B**) ABTS radical cation scavenging activity, (**C**) nitric oxide radical scavenging activity, and (**D**) reducing power of SCP-W, SCP-U, and SCP-M. SCP-W, hot water extraction of snow chrysanthemum polysaccharides; SCP-U, ultrasonic assisted extraction of snow chrysanthemum polysaccharides; SCP-M, microwave assisted extraction of snow chrysanthemum polysaccharides; BHT, butylated hydroxytoluene; *Vc*, vitamin C; the error bars are standard deviations; significant (*p* < 0.05) differences are shown by data bearing different letters (a–d); the statistical significances were carried out by ANOVA and Ducan’s test.

Pharmacological studies have shown that snow chrysanthemum tea possesses remarkable antioxidant activity [15]. However, the antioxidant activities of polysaccharides in snow chrysanthemum tea have seldom been investigated. Therefore, the antioxidant activities of snow chrysanthemum polysaccharides were measured, and the effects of the different extraction methods on their antioxidant activities were also investigated. The DPPH radical scavenging activities, ABTS radical scavenging activities, nitric oxide radical scavenging activities, and reducing power of SCP-W, SCP-U, and SCP-M were shown in Figure 5, respectively. Briefly, as shown in Figure 5A, the DPPH radical scavenging activities of SCPs exhibited a dose-dependent manner. The significantly (*p* < 0.05) highest DPPH radical scavenging activities were observed in SCP-M at different concentrations of 0.2 to 1.2 mg/mL, followed by the lower DPPH radical scavenging activities in SCP-U, and the lowest DPPH radical scavenging activities in SCP-W. The results suggested that the SCP-M extracted by the MAE method exhibited stronger antioxidant activities than that of SCP-W extracted by the HWE method and SCP-U extracted by the UAE method. The MAE method could be a good potential technique for the extraction of snow chrysanthemum polysaccharides with high antioxidant activities. In addition, the IC_50_ values of DPPH radical scavenging activities of SCP-W, SCP-U, and SCP-M were determined to be 1.702 mg/mL, 1.210 mg/mL, and 0.693 mg/mL, respectively, which confirmed that SCP-M exhibited the strongest antioxidant activities among SCPs extracted by different methods. The DPPH radical scavenging activity of SCP-M was similar with that of the polysaccharide fraction (KCCP fraction PII) isolated from snow chrysanthemum and the polysaccharide (CMJA0S2) isolated from *Chrysanthemum morifolium* flowers [1,11]. In addition, the DPPH radical scavenging activity of SCP-M was much higher than that of polysaccharides isolated from commonly consumed tea materials in China, such as dark tea (Fuzhuan brick tea) [57], oolong tea [58], and *Lycium barbarum* [59]. Moreover, the DPPH radical scavenging activity of SCP-M was similar with the positive control (BHT) at the concentration of 1.2 mg/mL, which suggested that SCP-M exhibited remarkable antioxidant activities.

Furthermore, as shown in Figure 5B, the ABTS radical scavenging activities of SCPs also exhibited a dose-dependent manner. Results showed that the significantly (*p* < 0.05) highest ABTS radical scavenging activities were also observed in SCP-M at different concentrations of 0.1 to 0.6 mg/mL, followed by lower antioxidant activities in SCP-U and the lowest antioxidant activities in SCP-W. Indeed, the IC_50_ values of ABTS radical scavenging activities of SCP-W, SCP-U, and SCP-M were determined to be 1.121 mg/mL, 0.614 mg/mL, and 0.299 mg/mL, respectively. Results further confirmed that SCP-M exhibited the strongest antioxidant activities among SCPs extracted by different methods. Moreover, compared with the BHT (IC_50_ = 0.095 mg/mL), SCP-M exhibited relatively weak ABTS radical scavenging activities.

As shown in Figure 5C, SCPs extracted by different methods also exhibited obvious scavenging activities on the nitric oxide radical in a dose-dependent manner. The IC_50_ values of nitric oxide radical scavenging activities of SCP-W, SCP-U, and SCP-M were determined to be 0.277 mg/mL, 0.128 mg/mL, and 0.105 mg/mL, respectively, which further confirmed that SCP-M exhibited stronger antioxidant activities than SCP-W and SCP-U. Indeed, the nitric oxide radical scavenging activities of SCP-M were extremely close to that of vitamin C (IC_50_ = 0.094 mg/mL), which indicated that SCPs exhibited remarkable nitric oxide radical scavenging activities.

As shown in Figure 5D, the significantly (*p* < 0.05) highest reducing power was also observed in SCP-M at different concentrations of 0.2 to 1.2 mg/mL, followed by a lower reducing power in SCP-U and the lowest reducing power in SCP-W. Although the reducing power of SCP-M was lower than that of BHT, it still reached 148.49 ± 3.68 μg Trolox/mg at the concentration of 1.2 mg/mL. All results suggested that SCPs could be one of the major contributors toward the antioxidant activities of snow chrysanthemum tea. Generally, the antioxidant activities of natural polysaccharides are correlated to their structure features, molecular weights, and compositional monosaccharides (uronic acids) [28,32,60,61]. It is estimated that the presence of electrophilic groups like keto or aldehyde in acidic polysaccharides facilitates the liberation of hydrogen from O–H bond, and these groups can improve the radical scavenging activities [62]. In the present study, the higher antioxidant activities (DPPH radical scavenging activities, ABTS radical scavenging activities, nitric oxide radical scavenging activities, and reducing power) observed in SCP-M might be partially attributed to its lower molecular weight and higher content of unmethylated galacturonic acids [63,64,65]. However, further purification, structural characterization, and in vitro and in vivo antioxidant activities of SCPs are required to reveal their structure–bioactivity relationships.

## 4. Conclusions

In this study, the optimal extraction conditions of UAE and MAE for the extraction of SCPs were obtained by response surface methodology. The effects of different extraction methods (HWE, UAE, and MAE) on the chemical structures and antioxidant activities of SCPs were investigated. Results showed that different extraction methods significantly affected the contents of uronic acids, molecular weights, molar ratio of constituent monosaccharides, and the degree of esterification of SCPs. In addition, SCPs exhibited remarkable antioxidant activities, which suggested that SCPs might be one of the major contributors toward the antioxidant activities of snow chrysanthemum tea. The high antioxidant activities observed in SCP-M extracted by the MAE method might be partially attributed to its low molecular weight and high content of unmethylated galacturonic acid. Results suggested that the MAE method could be an efficient technique for the extraction of SCPs with high antioxidant activity, and SCPs could be further explored as functional food ingredients and natural antioxidants for industrial application.

## Figures and Tables

**Table 1:** The Box–Behnken design with independent variables and observed values for ultrasonic-assisted extraction (UAE) and microwave-assisted extraction (MAE).

Runs	Levels of Independent Factors (UAE) ^a^			Extraction Yield %	Levels of Independent Factors (MAE) ^b^			Extraction Yield %
	*X*_11_ (%)	*X*_12_ (min)	*X*_13_ (mL/g)		*X*_21_ (W)	*X*_22_ (min)	*X*_23_ (mL/g)	
1	0 (60.0)	0 (20.0)	0 (40.0)	4.120	0 (480.0)	1 (8.0)	−1 (50.0)	3.824
2	−1 (40.0)	−1 (15.0)	0 (40.0)	3.037	0 (480.0)	0 (6.0)	0 (60.0)	4.258
3	1 (80.0)	−1 (15.0)	0 (40.0)	3.439	1 (560.0)	1 (8.0)	0 (60.0)	3.835
4	0 (60.0)	−1 (15.0)	−1 (30.0)	3.324	−1 (400.0)	−1 (4.0)	0 (60.0)	3.054
5	0 (60.0)	0 (20.0)	0 (40.0)	4.076	0 (480.0)	−1 (4.0)	1 (70.0)	3.436
6	0 (60.0)	−1 (15.0)	1 (50.0)	3.653	1 (560.0)	−1 (4.0)	0 (60.0)	3.449
7	1 (80.0)	0 (20.0)	1 (50.0)	3.762	0 (480.0)	0 (6.0)	0 (60.0)	4.244
8	0 (60.0)	1 (25.0)	1 (50.0)	3.715	0 (480.0)	0 (6.0)	0 (60.0)	4.227
9	−1 (40.0)	1 (25.0)	0 (40.0)	2.823	−1 (400.0)	1 (8.0)	0 (60.0)	3.499
10	0 (60.0)	0 (20.0)	0 (40.0)	4.088	0 (480.0)	1 (8.0)	1 (70.0)	3.713
11	0 (60.0)	0 (20.0)	0 (40.0)	4.062	0 (480.0)	−1 (4.0)	−1 (50.0)	3.139
12	1 (80.0)	1 (25.0)	0 (40.0)	3.682	−1 (400.0)	0 (6.0)	1 (70.0)	3.522
13	−1 (40.0)	0 (20.0)	−1 (30.0)	2.758	0 (480.0)	0 (6.0)	0 (60.0)	4.260
14	−1 (40.0)	0 (20.0)	1 (50.0)	3.317	−1 (400.0)	0 (6.0)	−1 (50.0)	3.512
15	0 (60.0)	0 (20.0)	0 (40.0)	4.033	1 (560.0)	0 (6.0)	1 (70.0)	3.921
16	0 (60.0)	1 (25.0)	−1 (30.0)	3.559	0 (480.0)	0 (6.0)	0 (60.0)	4.252
17	1 (80.0)	0 (20.0)	−1 (30.0)	3.624	1 (560.0)	0 (6.0)	−1 (50.0)	3.822

**^a^ UAE**: ***X*_11_**, ultrasound amplitude (%); ***X*_12_**, extraction time (min); and ***X*_13_**, ratio of water to raw material (mL/g). **^b^ MAE**: ***X*_21_**, microwave power (W); ***X*_22_**, extraction time (min); and ***X*_23_**, ratio of water to raw material (mL/g).

**Table 2:** The analysis of variance of the regression equation and coefficients of ultrasonic-assisted extraction (UAE) and microwave-assisted extraction (MAE)

Source ^a^	UAE					MAE				
	Sum of squares	d*f* ^b^	Mean square	*F*-value	*P*-value ^c^	Sum of squares	d*f* ^b^	Mean square	*F*-value	*P*-value ^c^
**Model**	2.98	9	0.33	121.60	<0.0001 **	2.50	9	0.28	541.05	<0.0001 **
***X*_11_ (*X*_21_)**	0.83	1	0.83	303.83	<0.0001 **	0.26	1	0.26	504.30	<0.0001 **
***X*_12_ (*X*_22_)**	0.013	1	0.013	4.85	0.0636	0.40	1	0.40	781.80	<0.0001 **
***X*_13_ (*X*_23_)**	0.17	1	0.17	64.22	<0.0001 **	0.011	1	0.011	21.30	0.0024 **
***X*_11_*X*_12_ (*X*_21_*X*_22_)**	0.052	1	0.052	19.18	0.0032 **	8.623 × 10^−4^	1	8.623 × 10^−4^	1.68	0.2362
***X*_11_*X*_13_ (*X*_21_*X*_23_)**	0.044	1	0.044	16.32	0.0049 **	2.016 × 10^−3^	1	2.016 × 10^−3^	3.92	0.0881
***X*_12_*X*_13_ (*X*_22_*X*_23_)**	7.483 × 10^−3^	1	7.483 × 10^−3^	2.75	0.1412	0.041	1	0.041	80.64	<0.0001 **
***X*_11_^2^ (*X*_21_^2^)**	1.11	1	1.11	408.70	< 0.0001 **	0.41	1	0.41	794.56	<0.0001 **
***X*_12_^2^ (*X*_22_^2^)**	0.42	1	0.42	154.82	<0.0001 **	0.96	1	0.96	1869.14	<0.0001 **
***X*_13_^2^ (*X*_23_^2^)**	0.16	1	0.16	59.70	0.0001 **	0.25	1	0.25	482.11	<0.0001 **
**Residual error**	0.019	7	2.722 × 10^−3^			3.596 × 10^−3^	7	5.138 × 10^−4^		
**Lack of fit**	0.015	3	4.968 × 10^−3^	4.79	0.0821	2.892 × 10^−3^	3	9.641 × 10^−4^	5.48	0.0670
**Pure error**	4.146 × 10^−3^	4	1.036 × 10^−3^			7.042 × 10^−4^	4	1.760 × 10^−4^		
**Correlation total**	3.00	16				2.51	16			

UAE, *R*^2^ = 0.9936, *R*^2^*_adj_* = 0.9855, coefficient of variation = 1.45%, and adeq. precision = 32.118; MAE, *R*^2^ = 0.9986, *R*^2^*_adj_* = 0.9967, coefficient of variation = 0.60%, and adeq. precision = 69.468. **^a^*X*_11_**, ultrasound amplitude (%); ***X*_12_**, extraction time (min); ***X*_13_**, ratio of water to raw material (mL/g); ***X*_21_**, microwave power (W); ***X*_22_**, extraction time (min); and ***X*_23_**, ratio of water to raw material (mL/g). **^b^***df*, the degree of freedom. **^c^** * significantly different (*p* < 0.05), ** extremely significantly different (*p* < 0.01).

**Table 3:** The chemical composition, molecular weights (*M_w_*), polydispersity (*M_w_*/*M_n_*), and constituent monosaccharides of SCP-W, SCP-U, and SCP-M.

	SCP-W	SCP-U	SCP-M
Extraction yield (%)	3.98 ± 0.22 ^a^	4.13 ± 0.24 ^a^	4.26 ± 0.21 ^a^
Total polysaccharides (%)	80.15 ± 0.54 ^a^	79.27 ± 0.42 ^a^	82.62 ± 1.09 ^a^
Protein content (%)	1.36 ± 0.11 ^c^	2.08 ± 0.19 ^b^	3.32 ± 0.26 ^a^
Total uronic acids (%)	40.88 ± 1.98 ^a^	34.45 ± 2.03 ^b^	30.45 ± 1.39 ^c^
Degree of esterification (%)	33.07 ± 2.17 ^a^	20.67 ± 1.92 ^b^	6.54 ± 1.55 ^c^
*M_w_* × 10^5^ (Da, error)	5.719 (± 0.86%) ^a^	4.768 (± 0.82%) ^b^	4.336 (± 0.84%) ^b^
*M_w_*/*M_n_* (error)	1.52 (± 1.22%)	1.81 (± 1.43%)	1.82 (± 1.68%)
Constituent monosaccharides and molar ratios			
Glucose (Glc)	1	1	1
Galacturonic acid (GalA)	6.37	2.75	2.04
Galactose (Gal)	5.22	2.55	2.41
Arabinose (Ara)	4.76	2.41	1.94
Rhamnose (Rha)	0.47	0.22	0.19
Mannose (Man)	0.19	0.30	0.22
Glucuronic acid (GlcA)	0.12	0.11	0.09
Xylose (Xyl)	0.05	0.04	0.05

SCP-W, hot water extraction of snow chrysanthemum polysaccharides; SCP-U, ultrasonic-assisted extraction of snow chrysanthemum polysaccharides; SCP-M, microwave-assisted extraction of snow chrysanthemum polysaccharides; the values represent mean ± standard deviation, and superscripts a–c differ significantly (*p* < 0.05) among SCP-W, SCP-U, and SCP-M; statistical significances were carried out by ANOVA, followed by Duncan’s test.
